# The Autistic Toe Walking: A Narrative Review for Interventions and Comparison with Idiopathic Toe Walking

**DOI:** 10.3390/children12091198

**Published:** 2025-09-08

**Authors:** Luiz Renato Agrizzi de Angeli, Bárbara Lívia Corrêa Serafim, Julio Javier Masquijo

**Affiliations:** 1 Department of Orthopedics and Traumatology, Hospital Israelita Albert Einstein, São Paulo 05652-900, SP, Brazil; 2 Department of Orthopedics and Traumatology, University of São Paulo, São Paulo 05402-000, SP, Brazil; barbara.serafim@usp.br; 3 Department of Orthopedics and Traumatology, Sanatorio Allende, Córdoba 5000, Argentina; jmasquijo@sanatorioallende.com

**Keywords:** Idiopathic Toe Walking, Autism Spectrum Disorder, neurodevelopmental delays, bracing, serial casting, surgical management

## Abstract

Background/Objectives: Idiopathic toe walking (ITW) is a diagnosis of exclusion in children who demonstrate a persistent toe-walking gait without an identifiable underlying neuromuscular or orthopedic pathology. The classification of toe-walking behavior (TWB) in children with Autism Spectrum Disorder (ASD) remains an area of debate, with some considering it a part of the broader ITW spectrum, while others view it as a distinct entity. Children with TWB associated with ASD (Autistic Toe Walking—ATW) present unique clinical challenges. This subgroup exhibits a higher prevalence of toe walking, and their gait patterns are often associated with underlying neurodevelopmental differences, frequently leading to increased resistance to conventional treatment approaches and higher rates of persistence and recurrence. This narrative review aims to summarize the available evidence on interventions for ATW, highlight differences compared to ITW and discuss implications for clinical practice. Methods: A literature search was performed, including articles that addressed interventions for toe walking in children with ASD. Results: The literature is limited and heterogeneous. Identified interventions include physiotherapy, orthoses, botulinum toxin injections, serial casting, and surgical procedures. Evidence of effectiveness is scarce, with most studies consisting of small case series. ATW differs from classic ITW in some aspects of pathophysiology and clinical presentation. Treatment decisions should balance potential benefits with risks, particularly regarding repeated anesthesia exposure during casting versus earlier surgical options. Conclusions: Evidence for managing ATW is limited. While comparisons to ITW may be useful, clinicians must recognize that they present distinct characteristics. Future research should focus on standardized definitions and controlled trials to guide management.

## 1. Introduction

Toe walking (TW) is a gait pattern characterized by the absence of heel contact with the ground during the stance phase. It can occur transiently in toddlers as part of normal motor development, but if it persists beyond the age of three years, it may indicate underlying conditions or functional adaptations. Idiopathic Toe Walking (ITW) is defined as a diagnosis of exclusion for individuals who exhibit toe walking behavior (TWB) for more than 50% of their walking time, without any associated neuromuscular causes [1,2,3,4]. The classification of children with TWB in the context of Autism Spectrum Disorder (ASD) remains a subject of debate in the literature. While some experts question whether these cases should be categorized as idiopathic, Bauer et al. recently proposed that TW associated with ASD is part of the broader ITW spectrum. We propose Autistic Toe Walking (ATW) to be categorized as a type of developmental non-neuromuscular toe walking. Throughout this review, we will adopt the terms TWB + ASD or ATW to avoid conceptual confusion and to highlight the need for tailored management strategies for this group of patients.

The prevalence of ITW in the general population may be as high as 5% during the early stages of ambulation, with spontaneous resolution reported in up to 79% of cases [5]. However, patients diagnosed with ASD demonstrate a significantly higher prevalence of PTW, ranging from 6.3% to as high as 62.9% [6], with most studies reporting an average prevalence of 20–40% and a high incidence of associated tight heel cords (12%) [7,8,9]. The exact etiology of PTW remains unknown; however, the literature consistently identifies associations between PTW and speech delays, sensory processing disorders (SPD), and other neurodevelopmental delays [7,10,11,12,13,14,15]. Given the high prevalence of these conditions in children with ASD, it is postulated that the increased prevalence of PTW in this population is linked to these factors; however, despite its frequency, there is no consensus on optimal management. Available approaches include physiotherapy, orthoses, botulinum toxin injections, serial casting, and surgical interventions, but the evidence remains limited and heterogeneous (Table 1) [4,8,15,16].

Zak et al. examined the effectiveness of surgical interventions for ITW and compared the outcomes in patients without additional diagnoses with those diagnosed with SPD and/or ASD. The study revealed that the rate of recurrence after surgical treatment was about five times higher in the latter group (24% vs. 5%) [23]. Consequently, TWB in the context of ASD (ATW) represents a more complex clinical entity, which requires tailored counseling for families to set realistic expectations regarding treatment outcomes and minimize recurrences and complications.

This review aims to summarize the available literature on interventions for ATW, clarify differences compared to ITW, and provide practical considerations for clinicians managing this challenging condition.

## 2. Methods

### 2.1. Literature Search Strategy

A comprehensive literature search was conducted in the databases of PubMed and Scopus, including articles published between 2005 and 2023. Search terms combined controlled vocabulary and free text related to toe walking, autism spectrum disorder, idiopathic toe walking, treatment, and intervention.

### 2.2. Inclusion and Exclusion Criteria

Studies were eligible for inclusion if they involved children or adolescents with a diagnosis of ASD; reported on interventions and other aspects for TW, and were written in English, Portuguese, Spanish, or French.

We excluded studies that focused exclusively on ITW without an ASD population; included adults only; or were opinion pieces or abstracts without original data.

### 2.3. Study Selection

Two independent reviewers (author 1 and author 2) screened all titles and abstracts for eligibility. Full texts were obtained for potentially relevant articles. Disagreements were resolved through discussion and consensus.

### 2.4. Data Extraction

Data were extracted using a standardized form, including: study design, sample size, population characteristics, type of intervention, follow-up period, and main outcomes.

### 2.5. Reporting

This review followed the main principles of the PRISMA 2020 statement, adapted to the context of a narrative review.

## 3. Results

### 3.1. Epidemiology

#### 3.1.1. Etiological Hypothesis

The etiology of ITW remains poorly understood [3,4,23,24]. However, several studies have identified potential associations with genetic predispositions, neurodevelopmental delays (such as speech delays and motor impairments), and SPD [9,13,24,25,26]. Children with ASD exhibit a significantly higher prevalence of PTW throughout their lives, further supporting the connection between motor sensory findings and TWB, suggesting that these factors may contribute to the development of the condition [7,8,9,27]. Nevertheless, it is essential to recognize that association does not equate to causation. The treatment of these associated conditions does not always result in the resolution of TWB, highlighting the complexity of the underlying mechanisms.

#### 3.1.2. Prevalence

Children with ASD have a significantly higher likelihood of developing PTW. The literature consistently supports this association, reporting a prevalence of TWB as high as 62.9% among patients with ASD [7,8,9,27], compared to an incidence of approximately 5% in the general population [5]. Additionally, the prevalence of gastrocsoleus contractures is notably higher in ATW, reaching up to 12% with variations observed between severe forms of ASD and milder presentations, such as those previously classified as Asperger’s syndrome [7,28]. One study demonstrated an odds ratio of 2.91 for the development of PTW and 2.28 for tight heel cords in children with ASD, compared to those with ITW and no other neurodevelopmental disorders [7].

Furthermore, the male-to-male ratio is significantly skewed in ATW cases (3:1), compared to an equal distribution (1:1) observed in ITW [8].

#### 3.1.3. Associations

Associations between TWB and ASD are well-documented in the literature, as evidenced by the significantly higher prevalence of TWB in individuals with ASD compared to those without associated disorders [7,8,9,27]. This relationship is primarily attributed to the high prevalence of comorbid conditions in ASD, such as speech delay, SPD, and other neurodevelopmental delays [9,10,13,24,25,26]. Interestingly, even in the absence of an ASD diagnosis, TW is frequently associated with these factors, underscoring their potential role in its etiology [10,13,24]. These connections are critical, as they suggest alternative pathways for PTW management beyond traditional interventions such as gait training and stretching exercises.

An important aspect of the link between ASD and abnormal gait patterns is their association with social impairment. A recent study involving 58 children with ASD identified correlations between impaired motor coordination and core symptoms of autism [9]. Abnormal gait patterns observed included PTW, flat-footed contact, left-right asymmetry, and increased step-to-step variability compared to controls. This highlights the potential role of gait abnormalities in influencing broader ASD manifestations and positioning gait as a promising target for integrated treatment approaches [4,27,28,29,30].

Valagussa et al. recently compared children with ASD exhibiting tip-toe behavior (ASD-TTB) to those without tip-toe behavior (ASD-NO-TTB) [14]. Both groups demonstrated SPD, but the ASD-TTB group exhibited a distinct pattern of “under-responsiveness/seeking sensation,” compared to the ASD-NO-TTB group. These findings underscore the importance of evaluating SPD not only in children with ASD, but also in other populations to optimize ITW treatment outcomes.

Furthermore, a study by Accardo and Barrow revealed an association between TWB + ASD and the persistence of components of the tonic labyrinthine reflex [11]. This observation suggests that certain motor behaviors in ASD may be linked to the retention of primitive reflexes, offering new insights into the underlying mechanisms of PTW in this population.

### 3.2. Natural History

The natural history ITW remains poorly understood, and even less is known about the natural evolution of ATW [5,7,29]. This knowledge gap is partly attributable to the multidisciplinary therapies often initiated immediately after ASD diagnosis, which likely influence the progression of TW. For instance, behavioral and SPD therapies may play a significant role in improving both TW and other ASD-related characteristics. Consequently, it would be unethical to withhold therapies from children with ASD to study the natural history of ATW. Given these ethical considerations, current evidence must be relied upon to understand the progression of ATW. Engström and Tedroff reported a general prevalence of ITW of 5%, with a spontaneous resolution rate of 59% by 5.5 years and 79% by 10 years of age [5]. However, Reinke’s deeper analysis of the data revealed that children with neurodevelopmental disorders, such as Attention Deficit Hyperactivity Disorder (ADHD) and ASD, had a significantly lower spontaneous resolution rate (59%) compared to children without such disorders (85%) [29]. This suggests that, even with multiple therapeutic interventions, children with ASD may exhibit lower spontaneous resolution rates for TW than untreated children without neurodevelopmental disorders.

A recent study found that children with TWB + ASD had a fourfold higher prevalence of PTW (6.3%) compared to those with ITW (1.5%) [6]. Barrow et al. also observed that the prevalence of ATW may decrease over time [7]. However, this reduction might reflect a cohort of adolescents with milder ASD presentations—potentially leading to delayed diagnoses—and a subsequently lower risk of developing PTW and gastrocsoleus contractures. Most evaluations in this study were performed on children aged 2–6 years, an age group with a higher likelihood of TW that could resolve spontaneously with time and ASD therapy. Despite these nuances, the authors reported a total PTW prevalence of 20.1% in children with ASD, which is four times higher than that reported by Engström and Tedroff [5]. Additionally, PTW in ASD was noted to persist longer, thereby increasing the likelihood of developing secondary gastrocsoleus complex contractures.

Another notable finding from Barrow et al. is the lower prevalence of ATW (20.6%) compared to earlier studies from over two decades ago, which reported rates as high as 62.9% [7]. This discrepancy likely reflects the evolving diagnostic criteria for ASD, which now include milder forms of autism such as cases without significant language impairments. These trends highlight the importance of severity classification of ASD when considering the incidence, natural history, and consequences of PTW.

Similarly, Leyden et al. reported a prevalence of TWB + ASD as high as 8.4% compared to 0.47% in patients with ITW [8]. Their study also demonstrated that children with TWB + ASD were three times more likely to require surgical intervention than those with ITW (3.3% vs. 1.2%). Furthermore, spontaneous resolution rates of ATW without specific TW treatment were substantially lower over a ten-year follow-up period (34.4% vs. 80.7% in ITW).

Collectively, these data suggest that ATW is associated with lower rates of spontaneous resolution, higher rates of gastrocsoleus contractures, an increased need for casting or surgical intervention, and a higher likelihood of recurrence following conservative management. Thus, the natural history of ATW appears to predict worse outcomes than ITW. These findings underscore the importance of vigilant monitoring and counseling pediatricians and parents regarding the elevated likelihood of requiring treatment in this population.

### 3.3. Evaluation

#### 3.3.1. History

We recommend using previously published questionnaires to exclude any secondary causes of TWB [24,30]. It is important to note that the Toe Walking Tool [30] refers to the presence of autism as a need for referral for further investigation, but does not exclude the diagnosis of ITW, as proposed by Bauer et al. [4]. Although the development of secondary deformities follows the same mechanism in ATW and ITW, it is important to acknowledge that they differ from each other in many aspects. This contrasts with primary motor neuromuscular diseases, such as Cerebral Palsy (CP) and Duchenne Muscular Dystrophy, where the underlying pathophysiology is different [4]. However, if any of the responses of the Toe Walking Tool [30] indicate a need for further investigation and a secondary motor cause is identified to explain the TWB, the child should be treated according to the actual diagnosis.

If no other condition is identified, the diagnosis of ITW is confirmed [4]. We recommend structuring the history-taking process with questions that help assess the severity of the condition and the likelihood of developing contractures that may require more invasive treatment. Based on current literature, we propose the following questionnaire for the initial evaluation of tip-toe behavior (TTB) [4,5,28,30,31,32,33]:

1. When did the child start toe walking?
2. Is the child on his/her toes [28]
  - While standing, walking, and running (TTB Class 1)?
  - While walking and running (TTB Class 2)?
  - Only while running (TTB Class 3)?
3. To what extent did the child walk on their shoes in the last week? Gradate in quartiles (0–25%; 26–50%; 51–75%; 76–100%)
4. Is the child able to stand up flatfoot without postural adaptations? (For example: pointing the feet outwards or leaning forward)
5. Is the child able to correct toe walking at his/her will?
6. Does the child feel any pain or activity restriction due to toe walking? Does the child present any traits of Sensorial Processing Disorders?
7. Does the child present with Motor Coordination Impairments?

The last two questions (7 and 8) might have a high rate of possible answers among children with ATW; nevertheless, it is important to determine whether these possible characteristics are being adequately addressed during their routine therapies.

These answers will provide baseline parameters that can be assessed in the future during follow-up for the analysis of treatment outcomes, helping the surgeon define the success or failure of the interventions applied [32].

#### 3.3.2. Physical Examination

The physical examination plays a crucial role in assessing the severity of TW and determining the need for intervention [4,28]. The attending surgeon must recognize that all morphological changes observed in children with ITW and ATW are directly related to the time spent walking on their toes—except in the rare cases of congenital TW, where patients are born with an Achilles tendon contracture [4]. In other words, the physical examination is going to confirm the veracity of the information gathered in the anamnesis. For example, a 5-year-old child who has been walking on their toes 90% of the time since the age of two is expected to present with gastrocnemius-soleus contracture, forefoot widening, forefoot callus formation, and calf muscle hypertrophy. This child is likely to be a strong candidate for intervention. Conversely, if physical examination does not reveal any of these findings, there is a significant chance that the actual percentage of time spent on toes has been overestimated. In these cases, observation may be the most appropriate management strategy.

#### 3.3.3. Inspection

**Figure 1:**
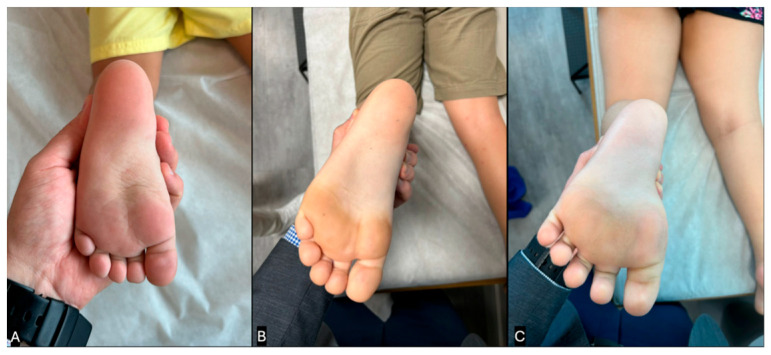
Clinical pictures showing the progression of morphological changes in the plantar aspect of the foot in children who toe walk. (**A**) Normal looking foot, with well-distributed forces between the hindfoot and forefoot. (**B**) Mild changes: forefoot callus, hindfoot narrowing, thin calcaneal skin, and external tibial torsion. (**C**) Severe changes: forefoot callus and widening, thin calcaneal skin, triangular shaped foot, and spread claw toes. (Clinical images from the personal files of Dr. Luiz de Angeli).

Inspection is a key component in the evaluation of TWB, as it provides valuable insights into both the severity of secondary morphological changes and the potential etiology of the condition [4,34]. If a child has spent a significant amount of time toe walking, progressive morphological changes are likely to develop in the following order (Figure 1):

- Forefoot callus;
- Forefoot widening;
- Hindfoot narrowing;
- Hindfoot pale and thin skin;
- Achilles tendon narrowing;
- Calf muscle hypertrophy;
- Reducible claw toes;
- External tibial torsion (increased thigh-foot angle or transmalleolar angle);
- Midfoot cavus or foot collapse into planovalgus.

**Figure 2:**
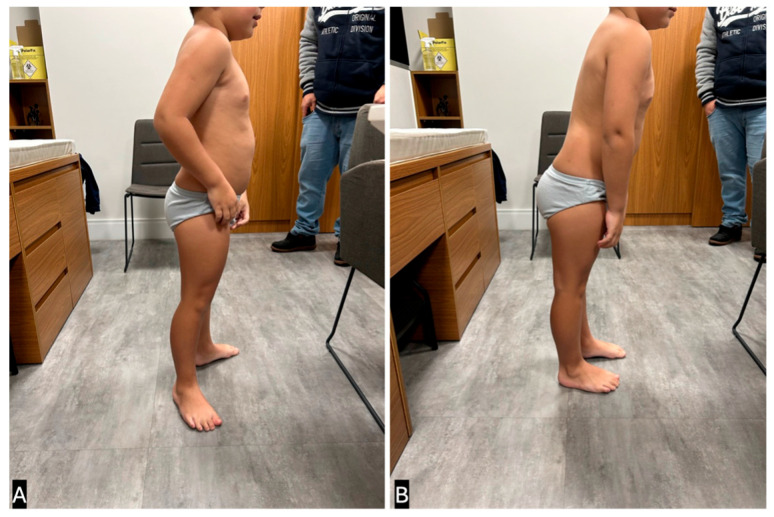
(**A**) Typical standing position of a patient who presented with a past neutral gastrocsoleus contracture. The patient abducts his legs and externally rotates his hips to achieve a comfortable position while standing, and is capable of completely touching the ground with both feet. (**B**) When asked to point his feet forward with his feet on the ground, the patient must lean forward to avoid falling back. This posture is highly predictive of severe gastrocsoleus contracture that requires casting or surgical treatment. (Clinical images from the personal files of Dr. Luiz de Angeli).

Notably, the combination of forefoot widening and hindfoot narrowing creates a triangular-shaped foot. Additionally, the senior author’s experience suggests that in the presence of severe gastrocnemius-soleus contractures, the transmalleolar angle is a more reliable indicator of external tibial torsion than the thigh-foot angle because it can be difficult to hold the subtalar joint in a neutral position when a tight Achilles tendon maintains the foot in varus. The examiner should also inspect the patient while standing. Patients with longstanding gastrocnemius-soleus contractures often widen their stance and externally rotate their feet while standing. This adaptation allows them to achieve a plantigrade position, even in the presence of severe contractures, by using external rotation to compensate for limited ankle dorsiflexion (Figure 2).

**Figure 3:**
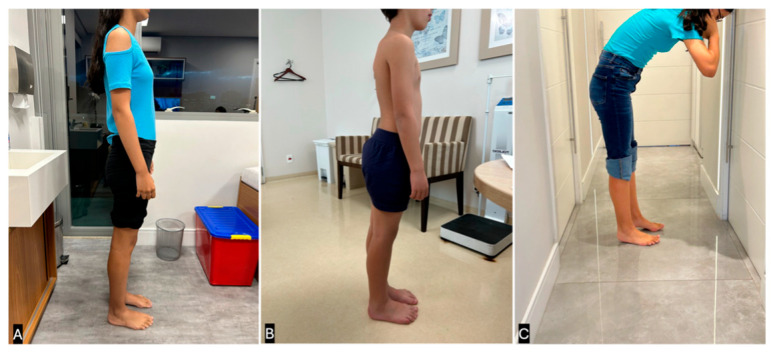
The Orthostasis Test. (**A**) Normal Posture: the patient can stand with her feet pointing forward with no difficulties. The patient is unlikely to have gastrocsoleus contracture. (**B**) Hyperlordosis Posture: the patient can only stand with his feet pointing forward with hyperlordosis compensation. The patient is likely to have a mild gastrocsoleus contracture and probably needs serial casting as the initial treatment. (**C**) Leaning-Forward Posture: the patient is leaning forward to try to stand with her feet pointing forward. The patient is likely to have severe gastrocsoleus contracture and probably requires surgery as the initial treatment. (Clinical images from the personal files of Dr. Luiz de Angeli).

An additional method for evaluating postural abnormalities related to gastrocnemius soleus contractures is what the senior author refers to as the “Orthostasis Test.” By positioning the patient in a sagittal view with their feet pointing forward, the examiner can observe three distinct postural patterns (Figure 3):

1. Normal Posture: The patient stands upright without trunk compensation, indicating that their ankle range of motion (ROM) is at least neutral or beyond neutral.
2. Hyperlordotic Posture: The patient voluntarily increases lumbar lordosis to maintain sagittal plane balance. This suggests a mild gastrocnemius-soleus contracture that prevents the ankle from achieving dorsiflexion beyond neutral position.
3. Leaning-Forward Posture: The patient leans their trunk forward to maintain balance and to avoid falling backward. This compensatory strategy is typically seen in cases of moderate to severe gastrocnemius-soleus contractures.

It is important to recognize that these compensatory postures and morphological changes indicate that PTW negatively affects the patient’s quality of life [31]. If any of these features are present, independently of the age of the child, the patient is probably not going to spontaneously resolve toe walking and might be a good candidate for intervention.

#### 3.3.4. Ankle Range of Motion Evaluation

The most used parameter to define management based on physical examination findings is the passive measurement of Ankle Range of Motion (ROM) [4]. The literature supports its use to determine the severity of TW and to measure outcomes after interventions [1,4,30,31,32,34,35,36,37,38,39,40,41,42,43,44,45,46].

It is essential to recognize that normative values for ankle ROM change over time according to the child’s age [47,48,49,50]. Although there is no consensus on the method of measuring ankle dorsiflexion across studies, a decrease in ankle ROM is expected throughout childhood into adulthood. We believe that the normative values presented by three of these studies [47,48,50] reflect the tendency of ankle ROM to decrease over time, with the knee both flexed and extended. However, the variations in the measurements published make it difficult to establish precise cut-off points to define whether ankle ROM is abnormal or normal within each age group. Additionally, variations in ankle ROM are likely influenced not only by age but also by genetic factors and foot morphology [47,48,49,50]. This observation highlights the importance of considering the full clinical presentation—especially functional limitations - of TW, rather than relying solely on ankle ROM values when deciding on management. For example, a 14-year-old male with ATW, who walks on his toes nearly 50% of the time and has neutral ankle dorsiflexion (zero degrees) with the knee extended, may not require specific treatment for TW. This is particularly true if his orthostasis test is normal and there are no secondary morphological changes in his limbs, as his ankle ROM is within normal values according to Liyanarachi et al. [48].

**Figure 4:**
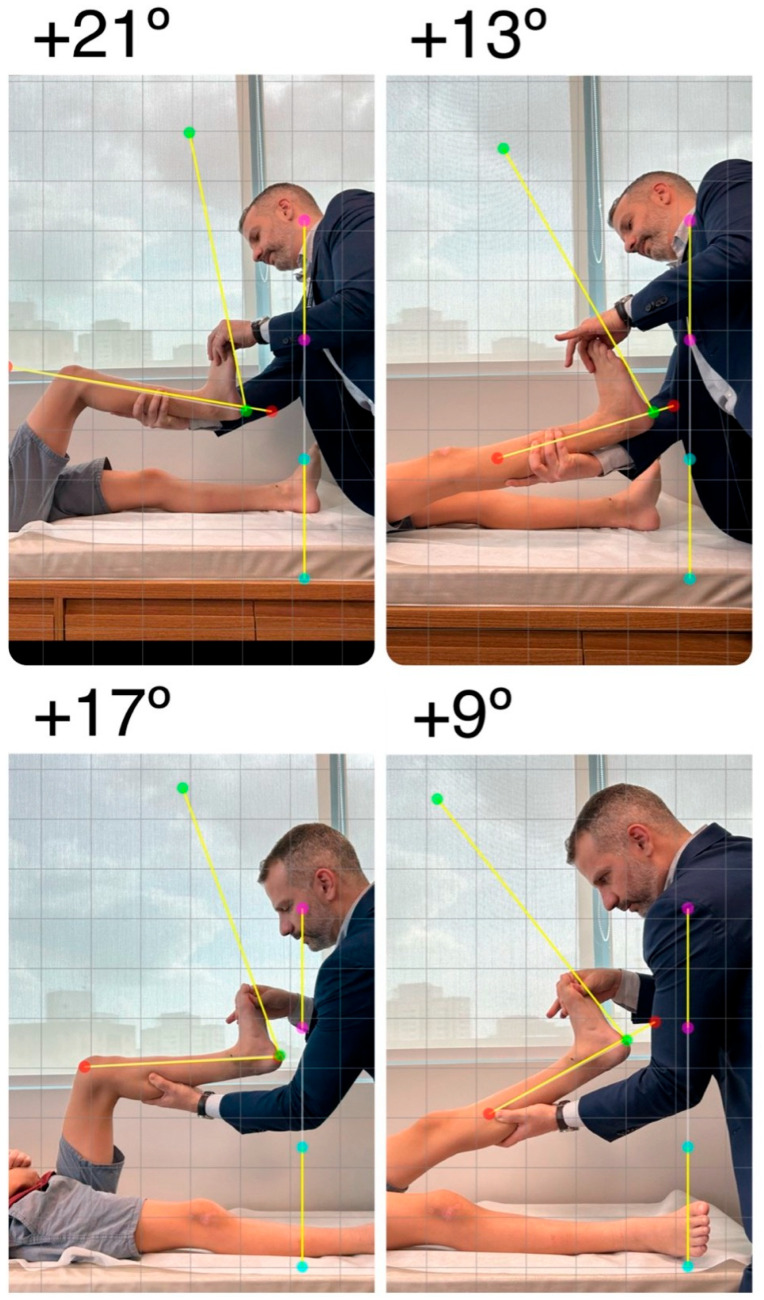
Clinical images showing the Silfverskiöld test were obtained. With the patient in a supine position, the foot is slowly and firmly dorsiflexed to its maximum degree, with the subtalar joint being held in the neutral position. Angle measurement is performed with a goniometer or by taking a photo in line with the axis of the ankle in the sagittal plane and measuring it digitally. The final dorsiflexion angle is obtained between the axis of the tibia and the lateral border of the foot. These steps should be repeated for each limb with the knee flexed and fully extended. (Clinical images from the personal files of Dr. Luiz de Angeli).

We recommend ankle dorsiflexion to be measured with the patient in the supine position and relaxed. The hindfoot should be held in a neutral position, and the forefoot slightly supinated to help bring the talus to a neutral position over the calcaneus [49]. The foot is then slowly and firmly dorsiflexed to its maximum degree, with the measurement taken using a goniometer or by photographing the ankle from the sagittal plane (Figure 4). The final angle is obtained between the tibia’s axis and the lateral border of the foot. These steps should be repeated for each limb, with the knee flexed to 90 degrees and fully extended—the Silfverskiöld test [51].

#### 3.3.5. Visual Gait Observation

It is important to recognize a key difference between primary motor neurologic gait patterns and ITW without severe contractures: idiopathic toe walkers might be able to correct their gait when concentrated or observed [35]. This is a limitation of both office gait observations and three-dimensional gait analysis (3DGA) for evaluating these patients. This capacity for gait correction may help in making the correct diagnosis, as good selective motor control leading to a heel-to-toe gait in the context of a history of toe walking suggests ITW or ATW.

The main differential diagnosis of ITW is mild diplegic CP [35,52]. The main differences between them are that children with ITW:

1. Can correct their gait pattern (at least partially when there are no severe contractures) when asked to;
2. Maintain their knees extended in the late swing phase and initial foot contact of the stance phase.

Although it can be difficult to observe the second characteristic in the office, knee kinematics should be assessed to differentiate ITW from mild diplegic CP. Children with ITW should display close-to-normal knee kinematics, while children with CP typically show a flexed knee pattern during the initial stance and late swing phases [35].

Evaluating a patient’s ability to correct their gait using the “Heel Walking Test” may be a good predictor of gastrocsoleus contracture [32,34]. By asking the child, “Can you try to walk on your heels for 10 steps with your feet in a typical distance apart, without changing your body position?”, the examiner can observe five distinct gait patterns [32]. The pattern should be documented for future follow-up comparisons.

#### 3.3.6. Three-Dimensional Gait Analysis

3DGA is the state of the art for evaluating gait patterns [29]. As mentioned earlier, 3DGA can help differentiate ITW from other conditions that manifest with TW. It is particularly useful for assessing knee kinematics, as ITW tends to show normal knee movement [35].

However, when dealing with ATW, several factors must be considered. First, many of these patients may be unable to follow instructions or allow the examiner to place markers on their skin, making 3DGA data collection difficult. Second, patients with ASD may have abnormal gait patterns that could slightly complicate the interpretation of 3DGA results in conjunction with TWB [9,53,54,55]. Children with ASD may present with developmental coordination disorders, leading to an asymmetric gait. Additionally, patients with ASD may exhibit more knee flexion during both the stance and swing phases and have a reduced ROM in all lower limb joints. This “clumsy” or “ataxic-like” gait with knee flexion may require further evaluation by other specialists to rule out primary causes of PTW. These abnormal findings may be more prevalent in children with severe ASD. Whenever there is doubt, a neurological evaluation should be performed to confirm or not the diagnosis of ATW and to proceed with the treatment plan.

#### 3.3.7. Follow-Up

We recommend following these children at least twice a year to measure outcomes and monitor progression. During these visits, we suggest using the Idiopathic Toe Walking Outcomes Proforma (iTWO Proforma) [32]. The main topics to be addressed will be listed in the Outcomes Evaluation section.

### 3.4. Management

#### 3.4.1. Therapy

Timely initiation of therapy is crucial for optimizing neurodevelopmental outcomes in children with ASD. Literature emphasizes that early diagnosis and intervention yield the best results [56].

While multidisciplinary therapies demonstrably benefit global neurodevelopment in children with ASD, specific evidence regarding their efficacy in resolving concurrent ATW is lacking [4]. Furthermore, the natural history of TWB within the ASD population is obscured, as affected children typically commence broad neurodevelopmental therapies soon after diagnosis. Consequently, the precise contribution of these general therapies to any observed improvement or resolution of TW remains unclear. Nevertheless, two key observations suggest that general ASD therapies may be insufficient for managing TWB in all affected children: the higher reported prevalence of PTW in the ASD population compared to neurotypical children, and the increased rates of surgical failure for TW correction in children with ASD compared to those with ITW [4,7,8,11,23].

Evidence evaluating therapies that specifically target TW in children with ASD is sparse. To date, the largest series examining directed physiotherapy for ATW, reported by Leyden et al. [8], found that 63.8% of patients treated solely with physiotherapy continued to toe walk within the first two years. Critically, the authors noted that this outcome could not be reliably differentiated from the condition’s natural history. Additionally, potential difficulties with patient compliance during physiotherapy sessions in this subgroup may limit its effectiveness as a standalone treatment [57].

Other nonsurgical interventions for ATW have been explored, primarily in case reports and limited cohorts. Studies utilizing auditory feedback mechanisms, such as audible conditioning stimuli (e.g., TAGteach™) or auditory speakers combined with habit reversal techniques, reported reductions in the percentage of time spent toe walking during therapy sessions [21,58]. Similarly, case reports described chiropractic management involving primitive reflex exercises [17] and behavioral reinforcement programs using discriminative stimuli [19] as potentially beneficial. However, these studies [17,18,19,21,22], were uniformly limited by their small sample sizes, evaluation predominantly within controlled therapy settings, lack of long-term follow-up data assessing functional gait improvements outside the clinic, and absence of replication in larger cohorts. Therefore, significant uncertainty persists regarding the optimal nonsurgical management strategy for ATW, underscoring the need for more rigorous, larger-scale studies with long-term functional outcome measures.

Similarly, Marcus et al. and Wilder et al. showed improvement in the gait pattern of three children each with TWB + ASD using GaitSpot Auditory Speakers and simplified habit reversal [18,22]. Nonetheless, these case reports [18,21,22,58] only evaluated patients during therapy sessions with no long-term follow-up and were not replicated later in larger cohorts.

Other case report studies have used different strategies to improve TWB + ASD. Shaw and Soto-Garcia reported a chiropractic management of an eight-year-old male patient with ATW based on the application of primitive reflex examination and motor coordination exercises, showing satisfactory results [17]. Hodges et al. found that a multiple schedule reinforcement program using a wristband as a discriminative stimulus could reduce toe walking in a 5-year-old male patient with ATW [19]. However, these case reports also did not evaluate long-term results in an outside-clinic environment and were not reproduced in larger cohorts [17,19].

##### Recommendations

Directed therapy treatment specifically for TWB in the context of ASD (ATW) is recommended under the following conditions:

1. The child is over three years of age;
2. Toe walking occurs during more than 50% of the observed gait time (time on toes, TTB > 50%);
3. This pattern has persisted for at least six months despite the child receiving standard multidisciplinary therapies for ASD [40];
4. There is no evidence of a fixed gastrocnemius or soleus contracture (i.e., passive ankle dorsiflexion passes neutral) (Figure 1) [48].

For children meeting criteria 1 and 3, but exhibiting TTB < 50% of the time and lacking contracture, continued observation alongside standard ASD therapies is advised. The development of morphological changes in the lower extremities, such as those previously described (e.g., forefoot widening and external tibial torsion), should prompt reassessment for potentially increasing the TTB percentage and consideration for initiating directed TW treatment (Figure 1).

Based on current evidence, isolated physiotherapy is not recommended as a primary treatment modality for ATW, given concerns regarding efficacy and potential compliance challenges [4,8,57,59]. Instead, a multidisciplinary approach to TW therapy is advocated. This team, potentially including physiotherapists, occupational therapists, and developmental specialists, should aim to identify and address potential underlying contributing factors such as SPD, persistence of primitive reflexes, or motor coordination impairments [11,40,53]. Potential therapeutic strategies within this framework include the following:

- Sensory integration therapies, potentially incorporating sensorial insoles;
- Auditory feedback techniques during gait training;
- Targeted motor coordination exercises;
- Interventions addressing persistent primitive reflexes;
- Behavioral modification techniques (e.g., multiple-schedule reinforcement).

Given the limited high-quality evidence supporting any single therapeutic modality for ATW, a pragmatic approach often involves combining several of these interventions tailored to the individual child’s profile to maximize potential benefits.

#### 3.4.2. Bracing

The use of Ankle Foot Orthosis (AFOs) for ITW in neurotypical children has been investigated, but findings are heterogeneous regarding AFO type, prescribed wear schedules, and reported outcomes [4,44,60,61]. For example, Bauer et al. suggested Posterior Leaf Spring Orthosis (PLSOs) as a treatment option for younger patients with ITW who retained more than 10° of passive ankle dorsiflexion [4]. Other authors have proposed night-time bracing, often used adjunctively following serial casting, surgery, or alongside other therapies, primarily to maintain ankle range of motion [1,4,16,34,40]. Comparative effectiveness studies remain scarce; notably, Herrin et al. reported better maintenance of outcomes after therapy cessation in patients using a foot orthosis compared to an AFO group in children with ITW [60].

Regarding ATW, there is currently no published evidence evaluating the efficacy of AFOs used as a standalone treatment. Existing reports have focused on the application of AFOs following serial casting to maintain correction. Manfredi et al. recommended night-time bracing after successful serial casting to preserve ankle dorsiflexion beyond neutral [16]. Barkocy et al., in a case report and small case series, described favorable results (maintenance of plantigrade gait) using day-time AFOs for approximately six months after completion of a serial casting protocol in patients with ATW [15,20]. Therefore, the current role of AFOs in ATW appears to be limited to preventing recurrence after initial correction achieved by other means, primarily by serial casting.

##### Recommendations

We do not recommend bracing as a solitary treatment for children with ATW. However, we suggest that the use of AFOs may be beneficial during the daytime for six months to a year following serial casting or surgical intervention. This approach aims to provide an extended period for neuromodulation of the gait pattern, to facilitate the regulation of sensory and motor coordination processes, potentially reduce relapse rates, and maintain the ankle dorsiflexion range of motion. We favor the use of PLSOs due to their streamlined profile, ease of integration with footwear, and ability to permit ankle dorsiflexion during the stance phase of gait. Based on the senior author’s experience, compliance among patients and families with night-time AFO use for ITW or ATW is poor, and this modality does not appear to contribute to gait neuromodulation processes. Therefore, we do not recommend the general use of nighttime bracing.

#### 3.4.3. Serial Casting

Serial casting is a widely utilized nonsurgical intervention to improve ankle joint dorsiflexion in children with ITW [4,15,16,20,38,39,41,42,43,46,57,62,63]. Although effective in increasing range of motion, there is conflicting evidence regarding the consistent resolution of ITW with serial casting protocols [4,42,43,63,64]. It is crucial to consider the resolution of the TW gait pattern, rather than solely the improvement in ankle dorsiflexion, as the primary outcome measure when evaluating treatment efficacy for ITW.

Few studies have specifically evaluated the use of serial casting in children with ATW [8,15,16,20,62]. Manfredi et al. published the largest case series to date, analyzing 22 children with ATW treated with serial casting [16]. Their protocol included the application of botulinum toxin A (BTX-A) days before the initial cast application, followed by nighttime bracing after cast removal. Cast changes were performed approximately every 14 days, with a total casting period not exceeding 30–40 days. They reported an improvement in ankle dorsiflexion; however, the recurrence rate, defined by the need to repeat the protocol, was high (50% in boys and 60% in girls). The percentage of time spent toe walking was not assessed as an outcome measure in this study.

Leyden et al. conducted a retrospective database review comparing treatment trends in 484 patients with ATW and 10,480 typically developing patients with ITW [8]. They observed a higher rate of serial casting use in the ATW group (7.4%) compared to the typical ITW group (3.6%). Recurrence rates were similar between the groups (ATW, 47.2% vs. ITW, 52.9%). Details regarding the specific casting protocols or the use of AFOs following treatment were not provided in this review.

Barkocy et al. presented a small case series (5 participants) and a case report examining the treatment of children with ATW using serial casting followed by daytime AFO use [15,20]. 3DGA was performed before and after treatment. The authors documented consistent improvement in ankle dorsiflexion during gait, as evaluated by sagittal kinematic analysis, following serial casting, and a subsequent six-month period of daytime AFO wear. Notably, in their case series of five patients [15], only two demonstrated normal ankle kinematics immediately after the serial casting phase, but all showed improvement after completing the six-month daytime AFO protocol. The AFOs were worn during waking hours and removed during sleep.

##### Recommendations

Serial casting is considered a primary nonsurgical intervention for addressing limited ankle dorsiflexion in children with PTW, with or without associated ASD [4,15,16,20,38,39,41,42,43,46,57,62,63]. Based on current evidence and clinical experience, the main indications for serial casting include:

- Failure to achieve satisfactory improvement after a 6 to 12-month trial of other nonsurgical modalities;
- Limited ankle dorsiflexion of greater than −10 degrees with the knee extended [4];
- Patient’s age up to 8 years [57].

Given that surgical intervention is typically not recommended for children without significant gastrocnemius-soleus contracture, the indications for serial casting may be extended to patients older than 8 years who present with an ankle dorsiflexion of 0 degrees or more.

**Figure 5:**
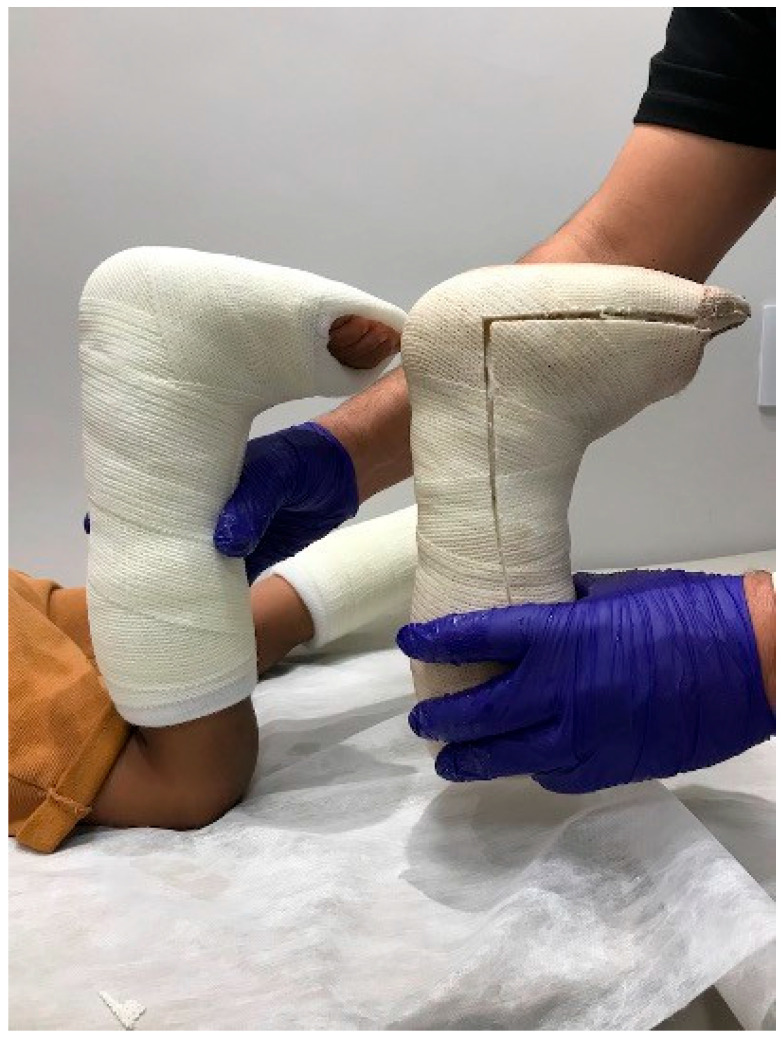
Image showing the difference in dorsiflexion achieved between the first serial cast session (right) and second application (left). The patients are stimulated to walk throughout the whole treatment period. (Clinical images from the personal files of Dr. Luiz de Angeli).

The senior author’s serial casting protocol involves performing cast changes every two weeks with the patient in a prone position and the knee flexed. Non-compliant patients might need cast changes and removal to be performed in the operating room under anesthesia, and this risk must be discussed with the parents before treatment initiation. For these patients, surgery must be considered, even if dorsiflexion is close to neutral. Casting continues until 10 degrees of ankle dorsiflexion is achieved with the knee extended, a method similar to that described by Bauer et al. [4]. Care is taken during dorsiflexion application to maintain a neutral hindfoot position to prevent subtalar joint eversion. In cases where the first cast achieves a neutral position that permits the patient to walk and stand comfortably, no cast change is necessary [16]. Waterproof fiberglass casting and padding materials are used, allowing for immediate weight-bearing after application (Figure 5). The minimum duration of the casting protocol is six weeks. Children are encouraged and permitted to bear weight and participate in activities such as walking, running, and jumping as tolerated while in the casts. Following the completion of the casting protocol, we recommend the use of PLSOs worn during the day for six months to a year [15,20].

We propose that this protocol can reduce the percentage of time spent TW by at least 50%. Achieving this level of improvement may help prevent progressive loss of ankle dorsiflexion, which often requires surgical management. Based on the senior author’s experience, it is uncommon for children with ATW to completely cease toe walking after serial casting. However, an outcome characterized by a reduction in TW time to less than 25% and the maintenance of greater than 10 degrees of ankle dorsiflexion with the knee extended should be considered a favorable result, as this status typically allows the child to avoid surgical intervention.

#### 3.4.4. Botulinum Toxin Type A

To date, there is no evidence that BTX-A treatment alters the natural history of PTW [36,37,38]. The effect of BTX-A, specifically in children with ATW, has not been extensively evaluated. One recent case series reported satisfactory results with the combined use of BTX-A and serial casting in children with ATW [16]; however, the absence of a control group in this study precludes drawing definitive conclusions regarding the specific contribution of BTX-A to the observed outcomes. Furthermore, a randomized controlled trial investigating the use of BTX-A in conjunction with serial casting for general ITW found no significant benefit from the addition of BTX-A [38].

##### Recommendations

Based on the limited and conflicting evidence and considering that the therapeutic rationale for BTX-A primarily targets spasticity, a condition not considered central to the pathogenesis of ITW or ATW, we do not recommend the use of BTX-A in the management of any type of PTW.

#### 3.4.5. Surgery

Surgical lengthening of the gastrocsoleus complex is a recognized treatment option for addressing TW and limited ankle dorsiflexion in children with ITW [1,4,8,63]. While high rates of TW resolution, potentially approaching 100% in some series, have been reported depending on the level of lengthening performed, limited data exist specifically for patients with ATW [1]. Studies evaluating surgical outcomes in the ATW population have suggested higher recurrence rates compared to ITW cohorts [8,23]. Zak et al. reported a close to five-fold increased likelihood of recurrence after surgery in children with ATW or SPD [23], and Leyden et al. observed a 75% recurrence rate in ATW patients versus 67.2% in ITW patients [8].

These higher recurrence rates in ATW cohorts may be influenced by factors beyond the diagnosis itself, including the specific surgical technique employed, which was not always specified in these studies [8,23]. Comparative studies using 3DGA have indicated superior outcomes with Zone III Achilles tendon lengthening (ATL), showing no recurrences in one series, in contrast to a 12% recurrence rate with Zone II ATL [1]. Furthermore, patients undergoing Zone III lengthening demonstrated a more favorable sagittal plane kinematic profile and preservation of muscle strength postoperatively. A recent series, although excluding patients with ASD, reported a 100% gain in dorsiflexion beyond neutral and complete resolution of the TW pattern in 19 patients over 10 years old treated with percutaneous Hoke ATL followed by a walking cast and rehabilitation [65]. The majority of studies reporting satisfactory surgical outcomes have described a Zone III ATL as the preferred procedure [1,65,66,67,68,69]. Currently, there is no strong evidence to suggest that the postoperative use of AFOs leads to superior results compared to not wearing them.

##### Recommendations

Surgical intervention is recommended for children over 8 years of age with ATW who present with less than 0 degrees of ankle dorsiflexion with the knee extended. Surgery is also indicated for any child presenting with 10 degrees or more of equinus contracture, regardless of age, or for patients who have failed at least one serial casting protocol and exhibit less than 10 degrees of ankle dorsiflexion with the knee extended.

The author’s preferred protocol is to perform a percutaneous Hoke ATL. This technique is favored even in patients with ankle dorsiflexion close to 0 degrees with the knee extended, based on evidence suggesting poorer outcomes with Zone II lengthening [1]. The wounds are closed with absorbable sutures. Postoperatively, a short leg walking cast is applied for six weeks, and immediate full weight-bearing is encouraged. To mitigate the risk of overlengthening, the cast is set between 0 and 5 degrees of dorsiflexion, even if greater dorsiflexion was achieved intraoperatively [66]. Following the casting period, we recommend the daytime use of PLSOs for a minimum of six months. This extended bracing period is particularly recommended for patients with ATW, as they may require more time to automate a heel-to-toe gait pattern. Non-compliant patients may require cast removal under anesthesia.

Based on the author’s clinical experience, children with ATW tend to tolerate surgical intervention better than serial casting. This improved tolerance may be attributable to the surgical release of the gastrocsoleus complex, which could alleviate the pain or discomfort associated with stretching post-procedure.

### 3.5. Outcome Evaluation

Treatment outcomes were evaluated at specific post-intervention time points to guide ongoing management. Consistent with criteria suggested by Bartoletta, we define a successful outcome at 1 year after initial intervention as the maintenance of TW time below 25% of the gait cycle and achievement of at least 10 degrees of ankle dorsiflexion with the knee extended [32,61]. To facilitate systematic monitoring, we recommend using the ITW Proforma at the initial visit and all subsequent follow-up appointments [32].

A satisfactory result is defined at 6 to 12 months after initial intervention as a reduction in TW time to less than 50% and maintenance of ankle dorsiflexion with the knee extended between 0 and 10 degrees. Patients achieving a satisfactory result require ongoing monitoring due to the potential risk of developing progressive contractures, which may necessitate further conservative measures or surgical intervention.

A poor result is characterized by the inability to achieve 0 degrees of ankle dorsiflexion with the knee extended, and PTW exceeding 50% of the gait cycle. Patients with poor results should be referred for reevaluation and consideration of alternative treatment strategies. To date, there is a paucity of studies that specifically evaluate optimal retreatment protocols after failed initial intervention in children with ITW. Given the increased likelihood of recurrence observed in patients with ATW, even after more invasive treatments [23], we strongly recommend considering surgical intervention following a failed trial of bracing or serial casting in appropriate candidates.

## 4. Discussion

This review highlights the limited and heterogeneous evidence available on ATW interventions. Most studies are small case series, with considerable variation in diagnostic criteria, intervention protocols, and follow-up periods [18,21,22].

It is essential to differentiate ATW from ITW, which occurs in children with normal neurological development. Although some clinical manifestations overlap, ATW presents with several distinct characteristics [4,34]. While ITW children may achieve a plantigrade stance when observed in cases without severe contractures, this is not always possible in cases of ATW. Moreover, in long-standing untreated ATW, persistent equinus may progress to severe midfoot deformities such as cavus or planovalgus. Recognition of this overlap is important, particularly because some children present the possibility of ASD and spastic CP, which drastically modifies the optimal treatment plan.

Non-surgical interventions, such as physiotherapy, orthoses, and botulinum toxin, generally provide limited and short-term benefits in ATW. Serial casting can improve ankle dorsiflexion in the short term, but the need for repeated anesthesia poses significant challenges in the ASD population [4,8,57,59]. This raises the question of whether earlier surgical intervention, such as ATL, may be preferable in selected cases [4,42,43,63,64]. Current evidence, however, is insufficient to establish standardized guidelines, and treatment decisions must be individualized.

For clinicians, the recognition that ATW is not idiopathic but rather related to a neurodevelopmental condition is fundamental. Management should balance potential functional gains against the risks of interventions, particularly repeated anesthesia. Early monitoring is essential to prevent fixed equinus contractures and secondary foot deformities.

The available studies are limited by small sample sizes, heterogeneous outcome measures, and a lack of randomized controlled trials. Standardized terminology and diagnostic criteria are needed. The authors believe future research should prioritize clear definitions and standardized terminology for ATW, controlled studies comparing conservative and surgical interventions, and long-term follow-up to assess recurrence and secondary deformities.

## 5. Conclusions

TWB is a frequent but poorly understood gait pattern in children with ASD. It must be recognized as a distinct condition from ITW, given the presence of a defined neurodevelopmental diagnosis as well as higher rates of PTW and treatment recurrence. Timely and appropriate intervention is crucial for improving gait mechanics, preventing secondary complications, and ultimately enhancing the child’s quality of life. Furthermore, clinicians should maintain a high index of suspicion for progression to fixed equinus and secondary deformities.

The current literature on interventions for ATW is scarce and heterogeneous. This review synthesizes the current knowledge and provides practical guidance to support clinical decision-making for TWB in the context of ASD.

High-quality research is essential to establish more definitive evidence-based treatment protocols tailored to the specific needs of this complex patient population.

## Figures and Tables

**Table 1:** Studies evaluating interventions for Idiopathic Toe Walking associated with Autism Spectrum Disorder.

Study	Participants	Age	Type of Intervention	Results	Conclusion
Manfredi et al. [16]	22 patients	From 4 to 15 years of age	“Cast and Go” protocol, including serial casting, botulinum toxin, orthoses, braces and support.	100% completion of protocol achieving neutral ankle position. However, the recurrence rate reached 54.5%.	A greater baseline ankle angle correlates with an increased need for plasters, and it emphasizes the secondary roles of botulinum toxin, orthoses, and rehabilitation in managing toe walkers.
Shaw et al. [17]	1 patient	8 years old	Cognitive-motor dual-tasking and primitive reflex integration exercises.	Significant improvement in gait mechanics.	Primitive reflex exercises could provide benefits to children with toe walking and ASD.
Barkocy et al. [15]	5 patients	Mean age of 9 years old	Serial casting followed by AFOs.	Resolution in all patients except the oldest one.	Serial casting, followed by AFOs were found to be effective to reduce toe-walking.
Wilder et al. [18]	3 patients	4 to 6 years of age	Use of auditory feedback with Gaitspot™ squeakers.	Reduction in toe walking for all participants.	Toe walking is reduced using auditory feedback produced by Gaitspot™ squeakers.
Leyden et al. [8]	484 patients with persistent toe walking + ASD	Under 19 years old	Physical therapy, serial casting and surgical correction.	63.8% of patients treated with physical therapy alone continued to toe walk; 47.2% with casting alone; 48.1% with physical therapy and casting; 75% with surgery continued to toe walk.	Poor rates of toe walking resolution in ASD patients; without intervention, 63.6% ASD patients toe-walked after 10 years, vs. 19.3% non-ASD.
Hodges et al. [19]	1 patient	5 years old	Evaluation of the use of a wristband as a discriminative stimulus and multiple schedules of reinforcement.	Effective decrease in toe walking.	The procedure was effective to decrease toe walking.
Barkocy et al. [20]	1 patient	7 years old	Serial casting followed by AFOs.	The participant increased passive dorsiflexion from −23° to −6° (left) and −18° to −8° (right). Range of motion improved, but not to normal values.	Child’s gait improved with serial casting followed or not by AFOs.
Persicke et al. [21]	1 patient	4 years old	Auditory reinforcement using a TAGteach™ tagger/clicker.	Decrease in toe-walking behavior.	TAGteach™ procedures could reinforce alternative appropriate behaviors and improve the child’s gait.
Marcus et al. [22]	3 patients	8 and 9 years old	Simplified habit reversal training with GaitSpot speakers and differential reinforcement.	Overall decrease in habituated toe-walking.	Behavioral methods may reduce habituated toe-walking for children with ASD.

## Data Availability

The original contributions presented in this study are included in the article. Further inquiries can be directed to the corresponding author.

## Abbreviations

ITW	Idiopathic Toe Walking
TWB	Toe Walking Behavior
ASD	Autism Spectrum Disorder
ATW	Autistic Toe Walking
TW	Toe Walking
PTW	Persistent Toe Walking
SPD	Sensory Processing Disorders
ASD-TTB	Autism Spectrum Disorder Exhibiting Tip Toe Behavior
ASD-NO-TTB	Autism Spectrum Disorder Without Tip Toe Behavior
ADHD	Attention Deficit Hyperactivity Disorder
CP	Cerebral Palsy
TTP	Tip-Toe-Behavior
ROM	Range of Motion
3DGA	Three-Dimensional Gait Analysis
AFO	Ankle Foot Orthosis
PLSO	Posterior Leaf Spring Orthosis
BTX-A	Botulinum Toxin A
ATL	Achilles Tendon Lengthening
